# ID 204 - Monitoramento do Horizonte Tecnológico (MHT) de Inibidores de Janus Quinase (JAKi) para o Tratamento de Vitiligo

**DOI:** 10.5327/2237-9622.2025.v34s1.204

**Published:** 2025-11-25

**Authors:** Renato Picoli, Renato Mantelli Picoli, Renata Ferreira, Júlia Lima, Pedro Antonio Barbosa, Aline Rocha, Natália Brandão, Luiza Costa, Hellen Miyamoto

**Keywords:** monitoramento do horizonte tecnológico, JAKi, vitiligo

## Abstract

**Introdução:**

O vitiligo é uma condição cutânea caracterizada pela perda de pigmentação devido à disfunção dos melanócitos. Embora não afete a expectativa de vida, o vitiligo não deve ser ignorado como uma condição meramente estética, pois pode levar à estigmatização e ao isolamento social. Apesar de ainda não estar completamente elucidado, o mecanismo geral de progressão do vitiligo está relacionado com a ação de citocinas, quimosinas e fatores de crescimento. As enzimas intracelulares da família JAK são responsáveis pela transmissão de sinais desses fatores e, dessa forma, sua inibição é um promissor alvo terapêutico. Este MHT visa identificar sistematicamente os JAKi em desenvolvimento para o tratamento do vitiligo.

**Método:**

As bases de dados ClinicalTrials.gov, WHO e Cochrane foram utilizadas para o levantamento de registros de ensaios clínicos. Foram utilizadas estratégias de busca com vocabulário controlado e seus respectivos sinônimos relacionados aos JAKi e a vitiligo. Não foram aplicadas restrições do tipo temporal, idioma ou status do estudo. Foram incluídos os ensaios clínicos de fase 2 e 3 e excluídos os estudos observacionais.

**Resultados:**

Cinco JAKi estão em desenvolvimento para o tratamento do vitiligo. Ritlecitinibe, um inibidor de JAK3/TEC, é indicado para pacientes com vitiligo não segmentar ativo e foi avaliado no ensaio de fase 2 (NCT03715829), que já está concluído com resultados, além de estar sendo estudado em dois ensaios de fase 3 (NCT06072183 e NCT05583526), ambos em fase de recrutamento. Baricitinibe, um inibidor seletivo e reversível de JAK1 e JAK2, é indicado para vitiligo não segmentar progressivo e foi avaliado no ensaio de fase 2 (NCT04822584), já concluído, mas sem resultados disponíveis. Upadacitinibe, também indicado para vitiligo não segmentar ativo, é um inibidor seletivo e reversível de JAK1, avaliado no ensaio de fase 2 (NCT04927975), que está concluído, mas ainda sem resultados. Cerdulatinibe, um inibidor de SKY/JAK, foi avaliado no ensaio de fase 2 (NCT04103060), já concluído, mas sem resultados disponíveis. Por fim, povorcitinibe, inibidor de JAK1, é indicado para vitiligo não segmentar e foi estudado no ensaio de fase 2 (NCT04818346), também concluído, mas sem resultados divulgados.

**Conclusão:**

O aspecto multifatorial da ativação e agravação do vitiligo torna complexo o desenvolvimento de um tratamento eficaz. O ritlecitinibe pode apresentar vantagens em relação aos tratamentos tópicos e à fototerapia na indução de repigmentação, em especial para casos de grandes áreas de lesão. O upadacitinibe, em ensaios de potência celular, demonstrou ser um inibidor mais potente da JAK1 em comparação com a JAK2 e a JAK3. Os estudos de cerdulatinibe e povocitinibe ainda aguardam publicação. Dentro do panorama do tratamento do vitiligo, os JAKi tornam-se medicamentos promissores. No entanto, os estudos clínicos ainda precisam avançar para estabelecer evidências claras sobre os reais benefícios clínicos dos JAKi para pacientes para o tratamento dessa condição.

